# 3D printing assisted MIPO for treatment of complex middle-proximal humeral shaft fractures

**DOI:** 10.1186/s12891-024-07202-w

**Published:** 2024-01-24

**Authors:** Chaoran Hu, Bing Qiu, Chaode Cen, Qin Luo, Yongfei Cao

**Affiliations:** Department of Orthopedics, The Beijing Jishuitan Hospital Guizhou Hospital, Guiyang, 550014 Guizhou China

**Keywords:** Middle-proximal humerus fracture, Minimally invasive percutaneous plate osteosynthesis, 3D printing, Open reduction and internal plating fixation, Preoperative simulation

## Abstract

**Background:**

This study was designed to explore the clinical efficacy of 3-dimensional (3D) printing assisted minimally invasive percutaneous plate osteosynthesis (MIPO) technique by comparing the clinical outcomes with traditional open reduction and internal plating fixation (ORIF) for treating complex middle-proximal humerus fractures (AO 12C fracture type).

**Materials and methods:**

The data of 42 participants who received a complicated middle-proximal humerus fracture from the beginning of 2018 to the end of 2022 were retrospectively analyzed. All patients were assigned to two groups: MIPO with detailed preoperative planning assisted by 3D printing technique (MIPO group), and traditional ORIF (ORIF group).

**Results:**

This study included 21 patients in the ORIF group and 21 patients in the MIPO group. All patients were followed-up for at least one year (mean: 16.12 ± 4.13 months), and no difference was observed in the range of shoulder joint motion (ROM), Quick Disabilities of the Arm, Shoulder and Hand (QuickDASH) scores and Constant scores between the two groups. However, the occurrence of complications (surgical incision site infection, implant loosening, bone nonunion and radial nerve palsy) in ORIF group was remarkably higher compared to the MIPO group. All the cases achieved bone union within the MIPO group. Significant differences were found in surgical time, intraoperative blood loss and fracture healing time between the two groups.

**Conclusion:**

Preoperative 3D printing assisted MIPO technique exhibits obvious advantages in high operational efficiency and low occurrence of complications, which is worthy of clinical application for treating complex middle-proximal humeral shaft fractures.

## Background

Fractures of the shaft of the humerus account for 3% of all fractures [1], and the middle segment is the most common site of fracture. The complex middle-proximal humeral fractures (AO 12C fracture type) are usually caused by high-energy injuries, accompanied by multi-level or comminuted fractures. These fractures are difficult to achieve good reduction and effective fixation. Open reduction and internal fixation (ORIF) is a common surgical approach employed for these complicated humerus fractures [2]. However, it may require extensive bone stripping, which can lead to possible complications.

Minimally invasive percutaneous plate osteosynthesis (MIPO) technique has emerged as an advanced surgical method that allows micromotion to induce osseous callus formation. It enables plate fixation without affecting the fracture sites, stabilizing the fractures and effectively reducing the risk of postoperative complications such as incision infection and bone nonunion [3]. However, this method has limitations in managing middle-proximal humeral fractures due to the complexity of the approach and the prolonged operative time, which can increase the incidence of complications.

Nowadays, 3-dimensional (3D) printing technology has been widely employed in the field of traumatic orthopaedics. It accurately reproduces the 3D physical model of the fracture site through radiographic projections using Computed Tomography (CT) data to assist clinicians in making detailed preoperative surgical plans. This includes predesigning reduction means, selecting appropriate internal fixation plate and performing necessary individualized shaping of the plate. These advancements greatly improve the precision of internal fixation, shorten operation time and reduce surgical difficulty. Therefore, 3D printing has become a commonly used adjunctive method for surgical treatment [4–6].

To address the shortcomings of ORIF and the limitations of MIPO, a new MIPO technique was developed. It involves detailed preoperative planning assisted by 3D printing to reduce surgical difficulty and operation time while ensuring surgical effectiveness and safety. In this research, we aim to explore the clinical efficacy of 3D printing-assisted MIPO technique for treating complex middle-proximal humerus fractures by comparing surgical outcomes and potential risks with traditional ORIF.

## Methods

### Patients

This research was approved by the institutional ethics committee of Beijing Jishuitan Hospital Guizhou Hospital. Informed consent was obtained from all participants. A retrospective analysis was conducted on 72 patients who underwent surgery for middle-proximal humerus fractures from the beginning of 2018 to the end of 2022 in our hospital.

The inclusion criteria were: (i) diagnosis of closed middle-proximal humeral shaft fractures via imaging examination; (ii) AO classification: all the fractures were 12C type; (iii) fracture time less than 3 weeks; and (iv) no neurovascular damage. Patients were excluded if they had (i) pathological fractures; (ii) combined with nerve injury; (iii) open fracture; (iv) a history of cognitive dysfunction or mental illness; (v) juvenile patients with unclosed epiphyses; (vi) combined with severe organic disorders; and (vii) follow-up less than 12 months. Of the 72 patients, 20 patients were excluded due to an ineligible fracture type, 3 patients were excluded because of combined with nerve injury, and 7 patients were excluded due to the follow-up time being less than 12 months. As a result, there were 42 cases of unilateral middle-proximal humeral fractures that met the inclusion criteria.

### Preoperative simulation

**Fig. 1:**
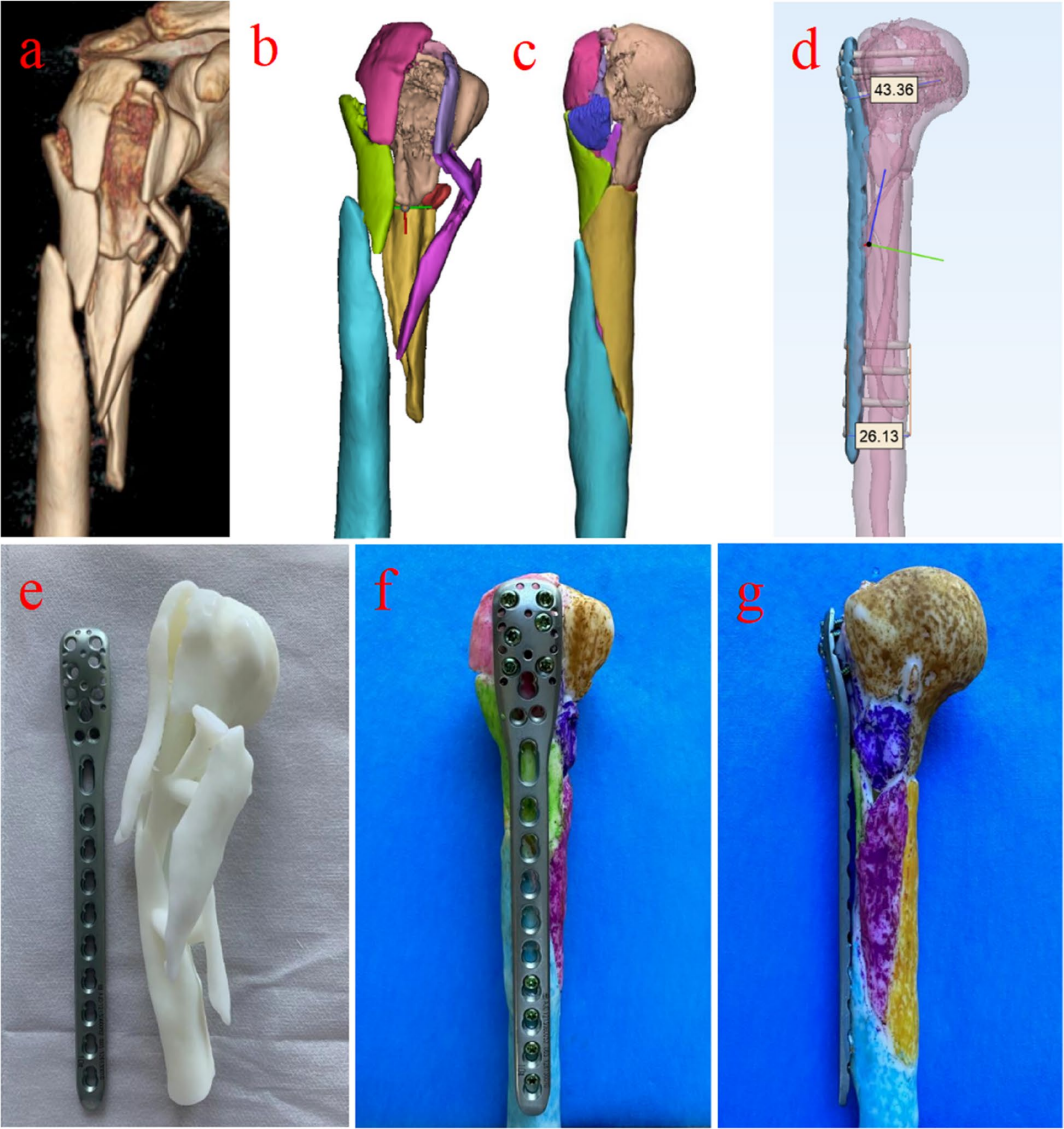
**a** Preoperative 3D CT image of the middle-proximal humerus fractures. **b** 3D digital model in Mimics software. **c** Virtual model for preoperative simulation. **d** The length measurement of the plate and the screws in the virtual model. **e** The 3D anatomical model of middle-proximal humeral fracture to determine the surgical plan and fracture fixation design. **f-g** 3D printed models for preoperative simulation

In the 3D printed MIPO group (MIPO group), CT image data obtained from the patients' affected humerus were stored in Digital Imaging and Communications in Medicine (DICOM) format, and images were reconstructed using the Mimics 21.0 software (Materialise, Leuven, Belgium). Then, we reconstructed the 3D digital images in the software, importing the data into the 3D-printing software in STL format. After forming a 3D digital model, we transferred it to a 3D printer (Lite 600, UnionTech, Shanghai, China) to reconstruct a full-scale model using photosensitive resin material. At the same time, the fracture models were imported into Materialise 3-Matic software (MATERIALISE LTD, Leuven, Belgium) to simulate surgery. Reduction of fractures included reduction of metaphyseal fracture, restoration of the medial column, and reduction of displaced greater tuberosity or lesser tuberosity. Subsequently, we selected the appropriate proximal humerus locking plate and screws, and determined whether the steel plate should be preflexed. Furthermore, once the fracture model was printed, surgeons were able to simulate the reduction in vitro and then validated on the 3D printed model. The chosen steel plate and screws were sterilized and stored for surgical use (Fig. 1).

### Surgical technique

All patients were treated consecutively by the same surgeon (Yongfei Cao from Beijing Jishuitan Hospital Guizhou Hospital).

The patients in MIPO group were placed in supine position under brachial plexus block with the upper limb on a table extension. The deltopectoral approach was used to make a 5-cm proximal incision, pull the cephalic vein to the inside for protection, and expose the fracture of middle-proximal humerus. The plate selected by 3D printed model was placed on the skin surface before operation to assess the length of the distal incision. To visualize the long head of the biceps brachii and the brachialis, the dissection was proceeded in line with the skin incision. Then, the musculocutaneous nerve was identified laying on the brachialis anterior muscle, the radial nerve and associated deep brachial artery were exposed in the brachialis posterior muscle and the lateral side of the middle humerus. The radial nerve was separated and preserved using a vascular sling. According to the 3D printing model, temporary fixation and reduction with Kirschner wires were performed on the main fracture blocks after traction and rotation to restore the humeral force line, keeping other small fracture blocks in place to avoid the exposure of the fracture area. For proximal humeral fractures (PHFs) with greater or lesser tuberosity, ligament lines should be placed at the insertion points of the subscapularis, supraspinatus, and infraspinatus in advance, tractioning the ligament lines and reduction with Kirschner wires. An extra-periosteal tunnel was generated from the distal to the proximal incision, the preselected proximal humerus locking compression plate (LCP) (XingRong Bolt®, Jiangsu, China) was inserted from the proximal end to the distal, closely attached to the humerus. These plates were used for all patients in the two groups. The top and medial of the plate are 5 ~ 8 mm and 2 ~ 4 mm distance from the humeral greater tubercle and the intertubercular sulcus, respectively. Once the reduction was satisfactory, a 3.5-mm diameter cortical bone screw was drilled proximally for reduction of the fractures. The number of screws relied on fracture morphology, the proximal locking screws were drilled up to 5 mm below the articular surface, and the distal were drilled sequentially, thus ensuring three bicortical locking screws with the largest distance between them based on the AO technical requirements [7]. C-arm fluoroscopy confirmed that the reduction and the position of the plate were satisfactory. For the 3-part or 4-part PHFs, fixation of the rotator cuff were provided through the holes around the steel plate. The shoulder was passively moved flexibly, and no impact was observed. The incision was completely stanched, rinsed, and then sutured without indwelling drainage tube.

The patients in ORIF group were treated with brachial plexus nerve and completed by the same surgeon. To achieve full exposure, ORIF were conducted using the traditional deltopectoral approach. The radial nerve and fracture area were completely exposed, the soft and hematoma tissues between the fragments were removed, and the fracture was anatomically reduced. The long version of the proximal humerus LCP was placed in the anterolateral humerus for fixation. C-arm fluoroscopy confirmed that the reduction and the plate’s position was satisfactory, and the shoulder was passively moved without any impact. After completely stanching, rinsing and closing the incision, the drainage tube was placed.

### Postoperative management and evaluation

All patients followed a rehabilitation program. After immobilizing the arms with a neck-wrist sling, the patients were asked to move the elbows one day after the operation. The shoulder was passively moved at one week after operation, active and active-assisted mobilization should be started at 6 weeks to restore shoulder range of motion (ROM) under the supervision of a physical therapist.

Intraoperative measurements included intraoperative blood loss, operative duration, fracture union time and hospital stay. The clinical outcomes were evaluated by the Constant score, QuickDASH score, the range of shoulder joint at the and the complications. The radial nerve injury, bone nonunion, surgical incision site infection and implant loosening were regarded as complications in this study. Nonunion was defined as the absence of clinical and radiographic evidence of union for up to nine months [8].

Follow-up studies were conducted at 1 month, 3 months, and then every 3 months after surgery until the plain radiographs of fracture healing was confirmed. Functional outcomes were evaluated at the end of the follow-up according to QuickDASH score, Constant score and the shoulder ROM.

### Statistical analysis

Statistical tests were conducted with SPSS v24.0. The measurement data are presented as mean ± standard deviation. Intergroup comparison was performed with T test. The counting data of the two groups were compared using a Pearson chi-square test. *P* < 0.05 was deemed statistically significant.

## Results

From January 2018 to December 2022, a total of 42 patients with middle-proximal humerus fractures were included in the study, 21 patients in the ORIF group and 21 in the MIPO group. The MIPO group contained 11 females and 10 males (mean age: 50.05 ± 12.75, range: 27–74). There were 13 cases on the right side, while 8 cases on the left side. Five of them had road accidents, while 16 fell or slipped. AO type: 12C1: 9 case; 12C2: 3 cases; and 12C3: 9 cases. The ORIF group contained 8 females and 13 males (mean age: 53.19 ± 12.72; range: 29–76). There were 10 cases on the right side, while 11 cases on the left side. Three cases had road accidents, while 18 fell or slipped. AO type: 12C1: 11 case; 12C2: 2 cases; and 12C3: 8 cases (Table 1).

**Table 1:** Patient demographics and fracture characteristics* (X* ± *S* or %*)*

	MIPO Group(*n* = 21)	ORIF Group(*n* = 21)	P
Age (year)	50.05 ± 12.75	53.19 ± 12.72	0.429
Sex			0.352
Male	10(47.6%)	13(61.9%)	
Female	11(52.4%)	8(38.1%)	
Fracture side			0.352
Left	8(38.1%)	11(52.4%)	
Right	13(61.9%)	10(47.6%)	
Mechanism of injury			0.432
Road accidents	5(23.8%)	3(14.3%)	
Falling down	16(76.2%)	18(85.7%)	
AO-OTA type			0.795
C1	9(42.9%)	11(52.4%)	
C2	3(14.3%)	2(9.5%)	
C3	9(42.9%)	8(38.1%)	

*MIPO *minimally invasive percutaneous plate osteosynthesis, *ORIF *open reduction and internal plating fixation

There were no significant differences in the general features between the two groups (*P* > 0.05). Compared to the ORIF group, the MIPO group had a lower intraoperative blood loss (141.90 ± 37.76 mL) and a shorter operation time (89.52 ± 6.69 min) (*P* < 0.05). The fracture healing time (13.38 ± 1.43 weeks) in MIPO group was significantly shorter than that in ORIF group (*P* = 0.000, < 0.05; Table 2).

**Table 2:** Comparison of the clinical outcomes between the two groups *(X* ± *S* or %*)*

	MIPO Group(*n* = 21)	ORIF Group(*n* = 21)	P
intraoperative blood loss(ml)	141.90 ± 37.76	261.90 ± 74.00	0.000
operation time(min)	89.52 ± 6.69	118.10 ± 11.99	0.000
hospital stay(days)	4.33 ± 0.66	4.67 ± 1.62	0.216
fracture healing time(weeks)	13.38 ± 1.43	15.06 ± 1.03	0.000
Complications			
radial nerve palsy	2(9.5%)	2(9.5%)	/
bone nonunion	0	4(19.0%)	/
implant loosening	0	0	/
incision infection	0	4(19.0%)	/
Complications incidence	9.5%	47.6%	0.006
QuickDASH score	15.79 ± 8.80	18.17 ± 10.42	0.429
Constant score	71.19 ± 10.27	69.86 ± 10.36	0.678
shoulder joint ROM (degrees)			
flexion	136.43 ± 15.26	129.29 ± 12.78	0.108
extension	43.10 ± 8.87	41.19 ± 8.65	0.485
abduction	131.43 ± 21.92	125.71 ± 13.81	0.319
external rotation	58.81 ± 8.50	55.48 ± 6.69	0.166
internal rotation	44.76 ± 10.18	41.67 ± 9.92	0.324

*MIPO *minimally invasive percutaneous plate osteosynthesis, *ORIF *open reduction and internal plating fixation, *ROM *range of motion, *QuickDASH* score the Quick Disabilities of the Arm, Shoulder and Hand Score System

**Fig. 2:**
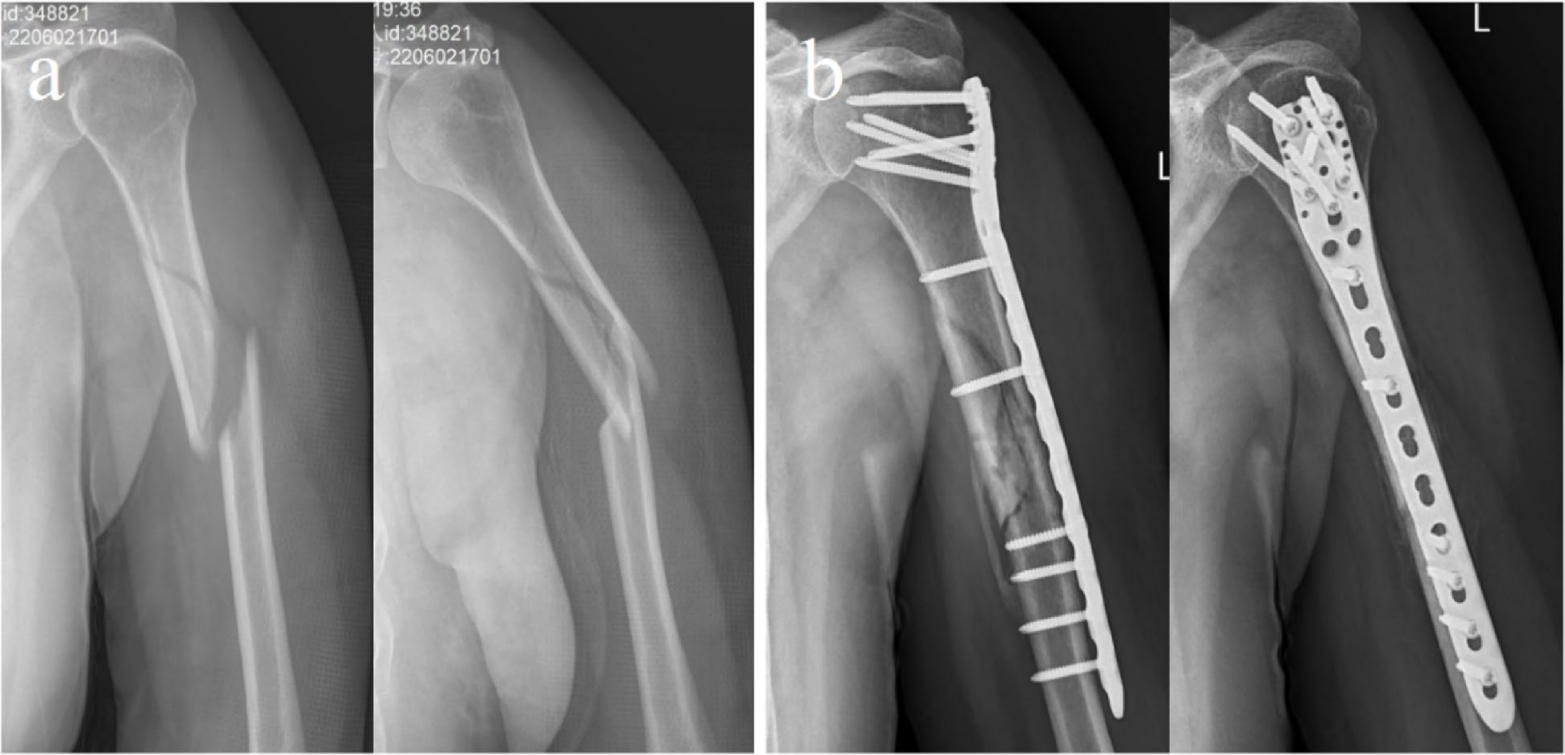
Case 1: a 64-year-old female, the AO/OTA classification was 12C1. **a** Preoperative X-ray radiograph of the middle-proximal humerus fractures. **b** The X-ray radiograph of full-length humerus 4 months after ORIF

**Fig. 3:**
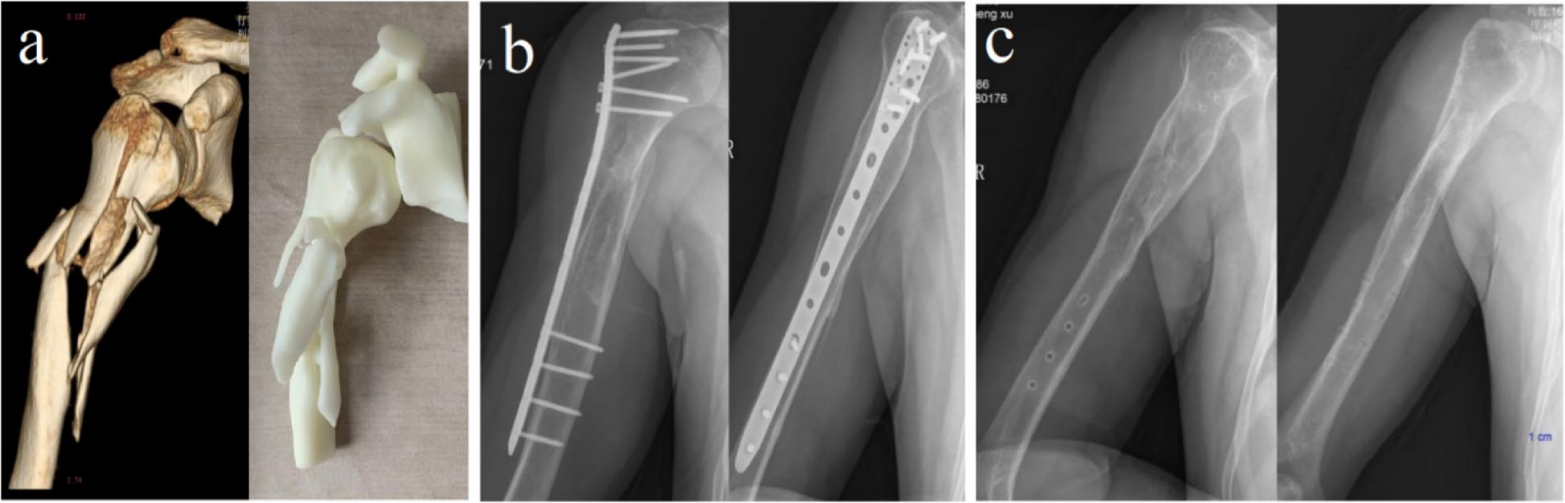
Case 2: a 41-year-old male, the AO/OTA classification was 12C13. **a** Preoperative 3D CT image and 3D anatomical model of middle-proximal humeral fracture. **b** The X-ray radiograph of full-length humerus 12 months after MIPO. **c** The X-ray radiograph of full-length humerus after removal of implants

**Fig. 4:**
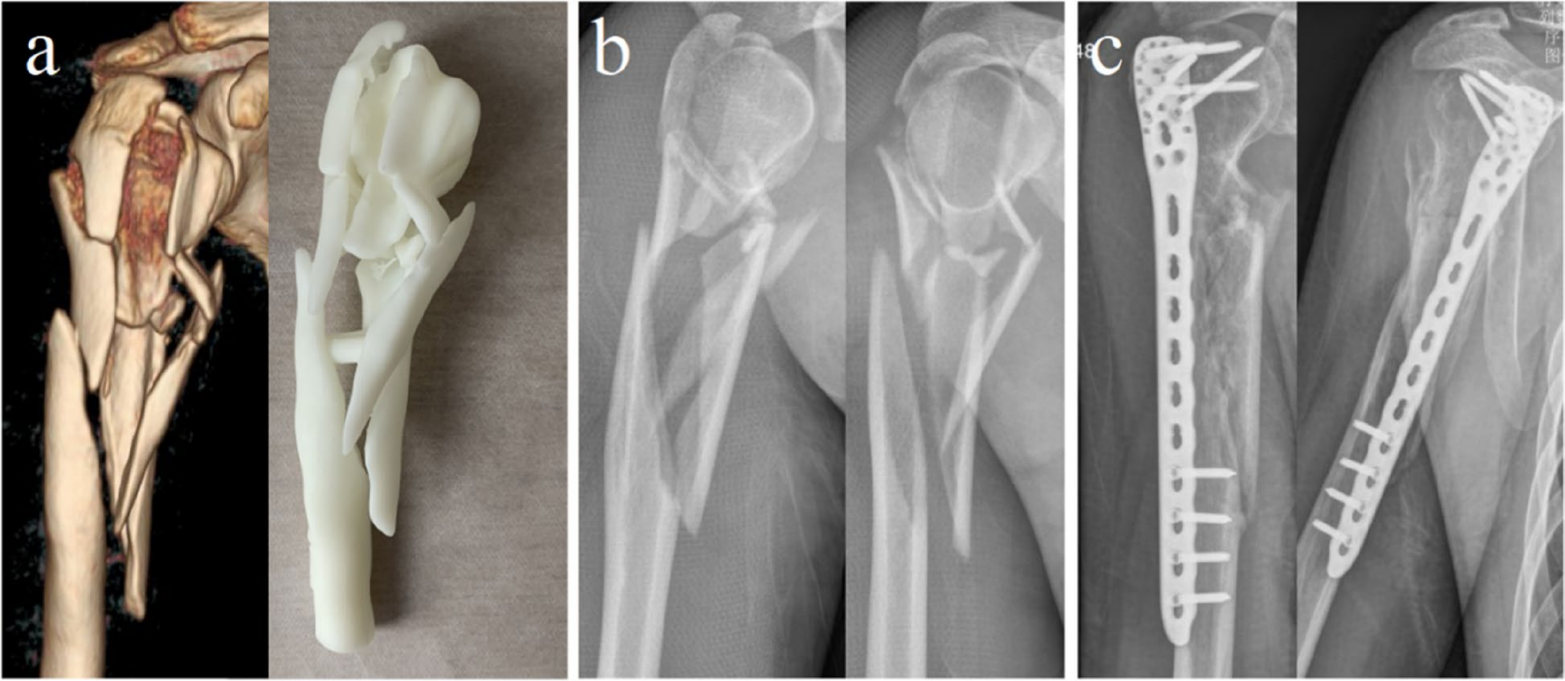
Case 3: a 33-year-old male, the AO/OTA classification was 12C13. **a** Preoperative 3D CT image and 3D anatomical model of middle-proximal humeral fracture. **b** Preoperative X-ray radiograph of the middle-proximal humerus fractures. **c** The X-ray radiograph of full-length humerus 4 months after MIPO

**Fig. 5:**
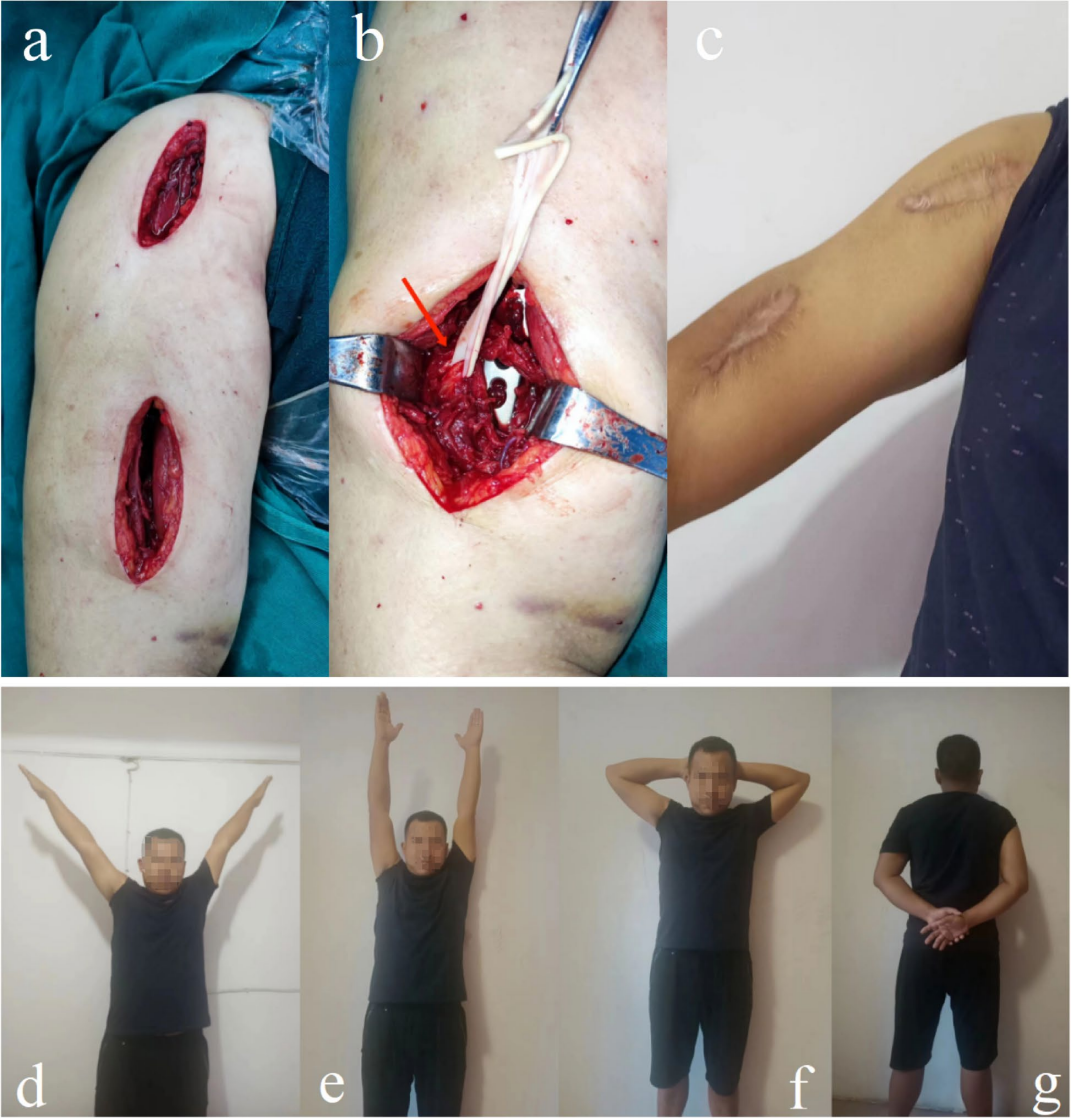
The selection and design of minimally invasive incision, and postoperative recovery of MIPO. **a** The appearance of an operative incision. **b** The radial nerve is exposed in the distal anterior incision intraoperatively with direct visualization and protection (marked by the red arrow). **c** The image of an postoperative incision 12 months after MIPO. **d** The shoulder joint recovered with satisfying function, abduction position, flexion position (**e**), supination position (**f**), and pronation position (**g**)

All patients were followed-up for at least one year (mean: 16.12 ± 4.13 months). Typical cases present the preoperative/postoperative follow-up imaging data of two groups for the treatment of complex middle-proximal humeral shaft fractures (Figs. 2, 3, 4, 5). Two cases of radial nerve palsy were observed in each of the two groups. There were four cases of incision site infection and bone nonunion in the ORIF group. The rate of complications was significantly higher in ORIF group than in MIPO group (*P* = 0.006 < 0.05; Table 2).

The average ROM of shoulder joint was flexion 136.43 ± 15.26° (ranging 110°–165°) vs 129.29 ± 12.78° (ranging 110°–155°), extension 43.10 ± 8.87° (ranging 35°–70°) vs 41.19 ± 8.65° (ranging 45°–70°), abduction 131.43 ± 21.92° (ranging 90°–150°) vs 125.71 ± 13.81° (ranging 90°–155°), external rotation 58.81 ± 8.50° (ranging 30°–65°) vs 55.48 ± 6.69° (ranging 20°–60°), and internal rotation 44.76 ± 10.18° (ranging 25°–60°) vs 41.67 ± 9.92° (ranging 25°–55°) between the two groups. No significant differences were observed in ROM, QuickDASH score and Constant score between the two groups (*P* > 0.05; Table 2).

## Discussion

In today's medical landscape, most humeral shaft fractures are more inclined towards surgical treatment as this facilitates a swifter reintegration of patients into society and their regular routines. Recently, many studies have reported on the treatment of middle-proximal humeral fractures. For example, Nicolaci [9] used a helical plate to treat middle-proximal humeral fractures, and suggested that stable fixation induced by the helical plate could reduce radial nerve distally and deltoid tuberosity proximally, thereby improving functional recovery after the surgical procedures. Wang et al. [8] demonstrated that both IMN and MIPO groups achieved good clinical outcomes (e.g., elbow and joint functions) with low complication rates in a total of 55 middle-proximal humeral fractures patients. The advantages of IMN include its minimally invasive surgical approach and reduction assistance without disturbing the fracture site, which in turn accelerates fracture healing. However, intramedullary fixation is also associated with a high incidence of complications, including nonunion, rotator cuff injury, acromion impingement and so on [8].

ORIF is a commonly practiced surgical approach for treating humeral shaft fractures. This method aims to achieve precise anatomical alignment but often involves substantial soft tissue dissection, which can lead to potential complications [10]. Recent studies have reported that MIPO technique can achieve good clinical effects for humeral shaft fractures [11]. In most cases, the MIPO procedure involves making an incision that is intentionally positioned away from the fracture site. This is done to protect the surrounding soft tissues and periosteum, thereby preventing direct exposure of the fracture. This approach supports the healing process by allowing the bones to unite without being overly exposed [12]. Aguado et al. [13] employed helical plates with MIPO technique, and the results indicated that satisfactory functional outcomes were obtained with a low risk of radial nerve injury, as evidenced by an average Constant-Murley score and shoulder abduction of 147° at the final follow-up. Therefore, this study aimed to compare the clinical outcomes of ORIF with MIPO technique for treating this type of fracture. However, achieving good reduction and effective fixation for AO type C middle-proximal humeral fractures can be challenging. This difficulty can lead to a higher likelihood of failed bone healing and surgical complications. Furthermore, the MIPO technique might extend the duration of the surgery due to its reliance on indirect realignment and the potential lack of proficiency, potentially necessitating a switch to the conventional ORIF approach. Such limitation restricts the suitability of the MIPO technique for addressing this specific fracture type. Therefore, to successfully complete the surgery with MIPO technique, we conducted a detailed preoperative plan with the assistance of 3D printing technology in MIPO group.

MIPO technique provides high biological stability and reduces the complications associated with ORIF [14, 15]. Large incisions can lead to soft tissue stripping, thereby increasing the risk of incision site infection and bone nonunion [16, 17]. MIPO technique, which only requires 2 small incisions, significantly reduces the risk of incision site infection and subsequent complications, along with less iatrogenic injury and shoulder discomfort. As a result, opting for an alternative approach would likely offer greater advantages in terms of restoring postoperative shoulder functionality [18]. In our work, the occurrence of complications (incision site infection, nonunion and implant loosening) was markedly lower in MIPO group than in ORIF group. However, there were no significant differences in ROM, Constant score and QuickDASH score between the two groups, indicating that both groups achieved favorable functional outcomes through early and professional rehabilitation training.

The utilization of 3D printing technology has enhanced the convenience of both diagnosis and treatment processes. It can construct initial fracture models using CT data based on radiographic projections, which helps visualize the fracture type and direction of displacement and provides an alternative option to complex surgical procedures for proximal-middle humeral shaft fractures [19]. Moreover, it facilitates comprehensive preoperative strategizing for the realignment of the fracture, leading to potential reductions in surgical duration and blood loss [20]. Furthermore, the utilization of 3D printing models brings a host of benefits to both patients and inexperienced surgeons. These models enable an intuitive grasp of the fracture's intricacies, an enhanced awareness of surgical challenges and associated risks, and an overall improvement in the dynamic between the healthcare provider and the patient [21].

Wang and colleagues [22] showed that pre-contouring plates with the assistance of 3D printing technology accurately fit the humeral shape, resulting in an average Constant score of 76.8 at the 12-month follow-up. Wu et al. [23] conducted a retrospective study on the treatment of PHFs. They divided patients into two groups based on whether 3D printing technology was used for preoperative simulation, and found that the simulation group had shorter operation times and less intraoperative bleeding. This suggests that 3D printing technology can improve clinical outcomes in the treatment of 3-part and 4-part PHFs. However, the majority of research endeavors have focused on utilizing 3D printing to aid in ORIF procedures for middle-proximal humeral fractures, and there exists a scarcity of investigations exploring the potential of 3D printing technology in the context of MIPO techniques. Given the complexity of such fractures, we utilized 3D printing for preoperative simulation, allowing for preoperative rehearsal of reduction and fixation on the 3D printing model. This approach aimed to improve surgical efficiency and reduce the need for transitioning to ORIF. Notably, all patients in the MIPO group successfully underwent the planned surgery without conversion to ORIF, and there were significant differences in fracture union time, intraoperative blood loss and operation time between the two groups. The MIPO technique proved to be a relatively safe and effective surgical approach for complex middle-proximal humeral fractures, potentially attributed to the precise preoperative planning enabled by 3D printing techniques.

In this study, two cases of radial nerve palsy occurred in each of the two groups. Iatrogenic radial nerve injury has been reported in 2.7%-20% of humeral shaft fractures, and is mainly associated with insufficient exposure and excessive traction [24]. In our recent study, we found that preoperative precise positioning using ultrasound and intraoperative pull methods reduced iatrogenic radial nerve injury, thereby increasing the efficiency of MIPO [25]. Additionally, the radial nerve passes through the posterior aspect to anterolateral at an average of 14 cm from the humeral lateral epicondyle [26]. Thus, the distal anterior approach can be used to directly visualize and protect the radial nerve. Considering the plate's insertion site, which was positioned a considerable distance from the radial nerve using the anterior approach, and taking into account our intraoperative visualization of the radial nerve in our study, the potential for inadvertent damage to nerves and blood vessels associated with the MIPO technique was notably reduced. The resolution of radial nerve palsy was evident two months post-surgery, underscoring the safety of the MIPO surgical approach.

Nevertheless, this study has several limitations. Firstly, 3D printing is merely an adjunctive technique to improve the efficiency of routine operations rather than a replacement. An inherent drawback of this method is its associated cost, which could potentially render it financially inaccessible for a considerable number of patients, thereby impeding its broad utilization [27]. Mandating preoperative 3D printing modeling for all patients is not a feasible proposition. Secondly, the 3D printing technique often requires 3–5 days to prepare a fracture model, rendering it time-intensive and unsuitable for scenarios necessitating urgent or emergency surgical interventions [28]. In addition, IMN is also a viable alternative for addressing this type of complex fracture. Hence, forthcoming research endeavors should encompass a comparative analysis between 3D printing-assisted MIPO with IMN, including the aspects of clinical efficacy and potential risks, in managing complex middle-proximal humerus fractures. Finally, the number of follow-up samples was relatively small, and the surgeon's proficiency had a significant influence on the study's outcomes. Therefore, augmenting the sample size in future investigations will bolster the validity and reliability of our findings.

## Conclusion

3D printing assisted MIPO technique has demonstrated its considerable value, effectiveness, and safety in managing complex middle-proximal humeral fractures, particularly with regards to operational efficiency. Integrating preoperative planning and surgical simulation through the aid of 3D printing technology contributes to a decrease in complications associated with internal fixation, enhances the precision of reduction, and ultimately betters the functional outcomes for patients. Consequently, its strong endorsement for clinical implementation in the treatment of complex middle-proximal humeral shaft fractures is well-founded.

## Data Availability

The datasets used and analyzed during the current study are available from the corresponding author on reasonable request.

## Abbreviations

3D: 3-Dimensional
ORIF: Open reduction and internal fixation
MIPO: Minimally invasive plate osteosynthesis
CT: Computed tomography
DICOM: Digital Imaging and Communications in Medicine
LCP: Locking compression plate
ROM: Range of shoulder joint motion
IMN: Intramedullary nailing
PHFs: Proximal humeral fractures
QuickDASH: Quick Disabilities of the Arm, Shoulder and Hand Score System

## References

[1] van de Wall BJM, Ochen Y, Beeres FJP (2021). Response to Yin et al regarding: "Conservative vs. operative treatment for humeral shaft fractures: a meta-analysis and systematic review of randomized clinical trials and observational studies". J Shoulder Elbow Surg.

[2] Maes V, Putzeys G (2021). One-year follow-up after treatment of proximal and/or middle one-third humeral shaft fractures with a helical plate: healing rates, complications and functional outcome measures. BMC Musculoskelet Disord.

[3] Xue Z, Jiang C, Hu C, Qin H, Ding H, An Z (2016). Effects of different surgical techniques on mid-distal humeral shaft vascularity: open reduction and internal fixation versus minimally invasive plate osteosynthesis. BMC Musculoskelet Disord.

[4] Li K, Liu Z, Li X, Wang J (2022). 3D printing-assisted surgery for proximal humerus fractures: a systematic review and meta-analysis. Eur J Trauma Emerg Surg.

[5] Honigmann P, Thieringer F, Steiger R, Haefeli M, Schumacher R, Henning J (2016). A simple 3-Dimensional printed aid for a corrective palmar opening wedge osteotomy of the distal radius. J Hand Surg Am.

[6] Bizzotto N, Tami I, Tami A (2016). 3D Printed models of distal radius fractures. Injury.

[7] Sermon A AO principles of fracture management. Acta Chirurgica Belgica. 2018 1–1.

[8] Wang Y, Chen H, Wang L (2021). Comparison between osteosynthesis with interlocking nail and minimally invasive plating for proximal- and middle-thirds of humeral shaft fractures. Int Orthop.

[9] Nicolaci G, Maes V, Lollino N, Putzeys G. How to treat proximal and middle one-third humeral shaft fractures: the role of helical plates [published online ahead of print, 2022 May 17]. Musculoskelet Surg. 2022;10.1007/s12306-022-00748-9. 10.1007/s12306-022-00748-9 35579822

[10] Kim JW, Oh CW, Byun YS, Kim JJ, Park KC (2015). A prospective randomized study of operative treatment for noncomminuted humeral shaft fractures: conventional open plating versus minimal invasive plate osteosynthesis. J Orthop Trauma.

[11] Shin SJ, Sohn HS, Do NH (2012). Minimally invasive plate osteosynthesis of humeral shaft fractures: a technique to aid fracture reduction and minimize complications. J Orthop Trauma.

[12] Lode I, Nordviste V, Erichsen JL, Schmal H, Viberg B (2020). Operative versus nonoperative treatment of humeral shaft fractures: a systematic review and meta-analysis. J Shoulder Elbow Surg.

[13] García-Virto V, Santiago-Maniega S, Llorente-Peris A (2021). MIPO helical pre-contoured plates in diaphyseal humeral fractures with proximal extension. Surg Technique Results Injury.

[14] Shetty MS, Kumar MA, Sujay K, Kini AR, Kanthi KG (2011). Minimally invasive plate osteosynthesis for humerus diaphyseal fractures. Indian J Orthop.

[15] Concha JM, Sandoval A, Streubel PN (2010). Minimally invasive plate osteosynthesis for humeral shaft fractures: are results reproducible?. Int Orthop.

[16] Rämö L, Sumrein BO, Lepola V (2020). Effect of surgery vs functional bracing on functional outcome among patients with closed displaced humeral shaft fractures: the FISH randomized clinical trial. JAMA.

[17] Rancan M, Dietrich M, Lamdark T, Can U, Platz A (2010). Minimal invasive long PHILOS®-plate osteosynthesis in metadiaphyseal fractures of the proximal humerus. Injury.

[18] Bogner R, Hübner C, Matis N, Auffarth A, Lederer S, Resch H (2008). Minimally-invasive treatment of three- and four-part fractures of the proximal humerus in elderly patients. J Bone Joint Surg Br.

[19] Chen C, Cai L, Zheng W, Wang J, Guo X, Chen H (2019). The efficacy of using 3D printing models in the treatment of fractures: a randomised clinical trial. BMC Musculoskelet Disord.

[20] Zhu D, Zhang Z, Zhang J (2020). The efficacy of 3D printing-assisted surgery in treating distal radius fractures: systematic review and meta-analysis. J Comp Eff Res.

[21] Tack P, Victor J, Gemmel P, Annemans L (2016). 3D-printing techniques in a medical setting: a systematic literature review. Biomed Eng Online.

[22] Wang Q, Hu J, Guan J, Chen Y, Wang L (2018). Proximal third humeral shaft fractures fixed with long helical PHILOS plates in elderly patients: benefit of pre-contouring plates on a 3D-printed model-a retrospective study. J Orthop Surg Res.

[23] Wu RJ, Zhang W, Lin YZ (2023). Influence of preoperative simulation on the reduction quality and clinical outcomes of open reduction and internal fixation for complex proximal humerus fractures. BMC Musculoskeletal Disord.

[24] Da Silva T, Rummel F, Knop C, Merkle T (2020). Comparing iatrogenic radial nerve lesions in humeral shaft fractures treated with helical or straight PHILOS plates: a 10-year retrospective cohort study of 62 cases. Arch Orthop Trauma Surg.

[25] Cen C, Cao Y, Zhang Y, Hu C, Luo C (2022). Preoperative position and protection of radial nerve by B-ultrasound combined with MIPPO for treatment of middle-inferior humerus fractures. J Orthop Surg Res.

[26] Benninger E, Meier C (2017). Minimally invasive lateral plate placement for metadiaphyseal fractures of the humerus and its implications for the distal deltoid insertion- it is not only about the radial nerve a cadaveric study. Injury.

[27] Wan SX, Meng FB, Zhang J, Chen Z, Yu LB, Wen JJ (2019). Experimental study and preliminary clinical application of mini-invasive percutaneous internal screw fixation for scaphoid fracture under the guidance of a 3D-printed guide plate. Curr Med Sci.

[28] Cook A, Baldwin P, Fowler JR (2020). Incidence of flexor pollicis longus complications following volar locking plate fixation of distal radius fractures. Hand (N Y).

